# SPL9 mediates freezing tolerance by directly regulating the expression of *CBF2* in *Arabidopsis thaliana*

**DOI:** 10.1186/s12870-022-03445-8

**Published:** 2022-02-02

**Authors:** Junli Zhao, Min Shi, Jing Yu, Changkui Guo

**Affiliations:** Laboratory of Plant Molecular and Developmental Biology, Zhejiang Agriculture and Forestry University, Hangzhou, 311300 China

**Keywords:** SPL9, CBF2, Freezing tolerance, Age, *Arabidopsis*

## Abstract

**Background:**

Freezing stress inhibits plant development and causes significant damage to plants. Plants therefore have evolved a large amount of sophisticated mechanisms to counteract freezing stress by adjusting their growth and development correspondingly. Plant ontogenetic defense against drought, high salt, and heat stresses, has been extensively studied. However, whether the freezing tolerance is associated with ontogenetic development and how the freezing signals are delivered remain unclear.

**Results:**

In this study, we found that the freezing tolerance was increased with plant age at the vegetative stage. The expressions of microRNA156 (miR156) and *SQUAMOSA PROMOTER BINDING PROTEIN-LIKE 9* (*SPL9*), playing roles in regulation of ontogenetic development, were induced by cold stress. Overexpression of *SPL9* (*rSPL9*) promoted the expression of *C-REPEAT BINDING FACTOR 2* (*CBF2*) and hereafter enhanced the freezing tolerance. Genetic analysis indicated that the effect of *rSPL9* on freezing tolerance is partially restored by *cbf2* mutant. Further analysis confirmed that SPL9 directly binds to the promoter of *CBF2* to activate the expression of *CBF2*, and thereafter increased the freezing tolerance.

**Conclusions:**

Therefore, our study uncovers a new role of SPL9 in fine-tuning *CBF2* expression and thus mediating freezing tolerance in plants, and implies a role of miR156-SPL pathway in balancing the vegetative development and freezing response in *Arabidopsis*.

**Supplementary Information:**

The online version contains supplementary material available at 10.1186/s12870-022-03445-8.

## Key message

SPL9 directly binds to the promoter of *CBF2* and activate *CBF2* expression to balance the freezing tolerance and plant development.

## Background

Plants frequently experience unexpected environmental changes, including drought and high or low temperatures, during their life cycle. The ability of plants to defend against these environmental changes is one of the major determinants of survival in natural populations. Plants therefore have evolved a plethora of sophisticated mechanisms to counteract the changing environment by adjusting their growth and development correspondingly; or plants select suitable regions to inhabit for reducing the freezing and other injuries [4]. It is well known that the interplay between ontogeny and abiotic stresses is of great importance in plants [6, 43]. Moreover, some stress responsive genes are highly upregulated in juvenile primordia in maize [37] for developmental priming and protecting against the damage by unexpected stresses. Evidences have shown that plants at the juvenile stage are more resistant to drought, heat stress and high salt stress [6]. MicroRNA156 (miR156) is the master regulator in regulation of developmental transitions by restraining SQUAMOSA PROMOTER BINDING PROTEIN-LIKE (SPL) in the posttranscriptional and translational levels [51, 52]. Overexpression of miR156 increases the juvenile stage with the elevated anthocyanin production in *Arabidopsis* and rice, and enhances abiotic stress tolerance by regulating the *SPL9* and *DIHYDROFLAVONOL-4-REDUCTASE* (*DFR*) expressions [6]. In addition, previous study shows that overexpression of miR156 in rice reduces the cold tolerance by repressing the expression of *OsSPL14* (or called *IDEAL PLANT ARCHITECTURE1* (*IPA1*)) [7]. However, the precise molecular mechanism of how miR156-SPLs pathway in an age-dependent manner modulates plant response to cold stress remains elusive.

Cold stress, including chilling stress (0 °C to 15 °C) and freezing stress (< 0 °C), is one of the major environmental factors inhibiting plant growth and development, and even causing significant crop losses [40]. Under cold stress, the plasma membrane of plant is damaged with increased electrolyte leakage. Moreover, the number of stomata is associated with the cold-tolerance of varieties in plants: the sensitive cultivars and genotypes have the highest number of stomata [3]. Deciphering the mechanisms underlying the response to cold stress helps to accelerate the breeding of cold-tolerant varieties in plants. In recent years, many differently-expressed genes that play roles in the cold responsive network in plants have been identified by transcriptomic analysis. Of them, some cold-induced genes are enriched in the responses to abiotic stresses; some cold-repressed genes are enriched in the regulation of transcription, and response to hormone stimulus [16, 38, 41, 54]. In addition, some flowering genes, including *FLOWERING LOCUS T* (*FT*) and *SUPPRESSOR OF OVEREXPRESSION OF CO 1* (*SOC1*), are increased by cold to control flower development [21, 39], indicating that some developmental genes function in cold response to balance the developmental processes under cold condition by interacting with cold-responsive genes.

Some studies have indicated that the C-REPEAT BINDING FACTOR/DRE BINDING FACTOR1 (CBF/DREB1) transcription factors play critical roles in cold tolerance response in plants. CBFs are directly downstream of ICEs (INDUCER OF CBF EXPRESSION) and directly bind to the C-repeat (CRT)/dehydration-responsive element (DRE; G/ACCGAC) of the Cold-Regulated (COR) genes, known as the “CBF regulon”, to activate their expressions for further increasing the cold tolerance [5, 29, 46]. In *Arabidopsis*, three *CBFs*, including *CBF1*, *CBF2*, and *CBF3* (also called *DREB1b*, *DREB1c*, and *DREB1a*, respectively), tandemly clustered in an 8.7-kb region of the fourth chromosome, are strongly and transiently up-regulated within 1 ~ 3 h after cold treatment, and thereafter their expressions are rapidly declined [18, 33, 34, 44, 47]. However, their expression patterns are different: *CBF1* and *CBF3* genes are mainly expressed in roots, hypocotyls and cotyledons, whereas *CBF2* is expressed in hypocotyls, cotyledons and leaves under normal condition; when the plants are treated with cold, *CBF1*, *CBF2* and *CBF3* genes are all expressed in leaves, but *CBF2* is also expressed in shoot stems [36]. Overexpression of *CBFs* leads to dwarf plants and enhanced cold tolerance [17, 23]. Since the three CBF proteins exhibit very high sequence similarity, they possibly have functional redundancy. *cbf1* and *cbf3* single mutants have slightly greater freezing tolerance than the wild type, whereas the mutation in *CBF2* gene increases sensitivity to freezing with lower survival rate and greater ion leakage [54]. These results show that CBF2 is more important than CBF1 and CBF3 in the regulation of freezing tolerance.

The precise expression regulation of CBFs is of key importance for maintaining the balance between cold tolerance and plant growth. Therefore, how CBFs are regulated in response to cold tolerance and how CBFs regulate cold responsive genes expression are widely studied. A series of regulators, including INDUCER OF CBF EXPRESSION 1/2 (ICE1/2) [14, 29], MYB15 [2, 50], ZINC FINGER OF ARABIDOPSIS THALIANA 12 (ZAT12) [47], CALMODULIN-BINDING TRANSCRIPTION ACTIVATORS (CAMTA) [11], PHYTOCHROME-ASSOCIATED PROTEINS (PIFs) [26], CESTA (CES) [13], ETHYLENE-INSENSITIVE 3 (EIN3) [42] REVEILLE4/LHY-CCA1-Like 1 (RVE4/LCL1) and RVE8/LCL5 [27] that control the expression of *CBF* genes upon cold stress have been identified [25, 26]. ICE1 plays key roles in cold stress response by directly binding to the promoters of *CBFs* to control their expressions. The kinase OPEN STOMATA 1 (OST1) phosphorylates and stabilizes ICE1 to facilitate the expression of the *CBF* genes [9]; in addition, OST1 enhances the interaction between BASIC TRANSCRIPTION FACTOR 3 (BTF3s) and the CBFs for the stability of CBF proteins under cold stress [10]. Yet the protein kinase BRASSINOSTEROID-INSENSITIVE2 (BIN2) phosphorylates ICE1 for promoting ICE1 degradation by boosting the interaction between ICE1 and the E3 ubiquitin ligase HIGH EXPRESSION OF OSMOTICALLY RESPONSIVE GENE 1 (HOS1), and thereby downregulating *CBF* gene expression [53]. BRASSINAZOLE-RESISTANT 1 (BZR1) and its closest homolog BRI1-EMS-SUPPRESSOR 1 (BES1), downstream of BIN2, positively regulate plant cold tolerance [32]. Further studies show that BZR1 directly binds to the promoters of *CBF1* and *CBF2* genes in vivo to activate their expressions in *Arabidopsis* [32]. CIRCADIAN CLOCK-ASSOCIATED 1 (CCA1) and LATE ELONGATED HYPOCOTYL (LHY) directly bind to the promoters of *CBF1*, *CBF2*, and *CBF3*, and have a direct role in their circadian regulation [12]. Moreover, CBF2 negatively regulates the expression of *CBF1* and *CBF3* [35]; and CBF1 and CBF3 also negatively affect the gene expression of *CBF2* [54].

CBFs are the key factors in the complex cold-responsive network, and are also major players in determining the cold tolerance of plants. Moreover, the *cbf* triple mutants exhibit smaller rosette leaf number and size and lower fresh weight than the wild type [54], suggesting that CBFs function in the balance of plant development and cold-tolerance. To date, miR156-SPLs pathway also plays critical roles in plant development and cold response; and whether there is the interaction between CBFs and miR156-SPLs pathway in regulating cold tolerance is not reported. In this study, we provide evidence that the capacity of freezing tolerance in plants is increased with plants age; and SPL9 directly binds to the promoters of *CBF2* to promote *CBF2* expression for enhancing freezing tolerance. Overexpression of *SPL9* (*rSPL9*) reduced the juvenile stage, and conferred enhanced freezing tolerance. SPL9 acts as a direct transcriptional activator to promote the expression of *CBF2*. Therefore, SPL9 constitutes a new hub to balance the development and cold tolerance in plants.

## Results

### Age-dependent freezing tolerance during vegetative development

**Fig. 1:**
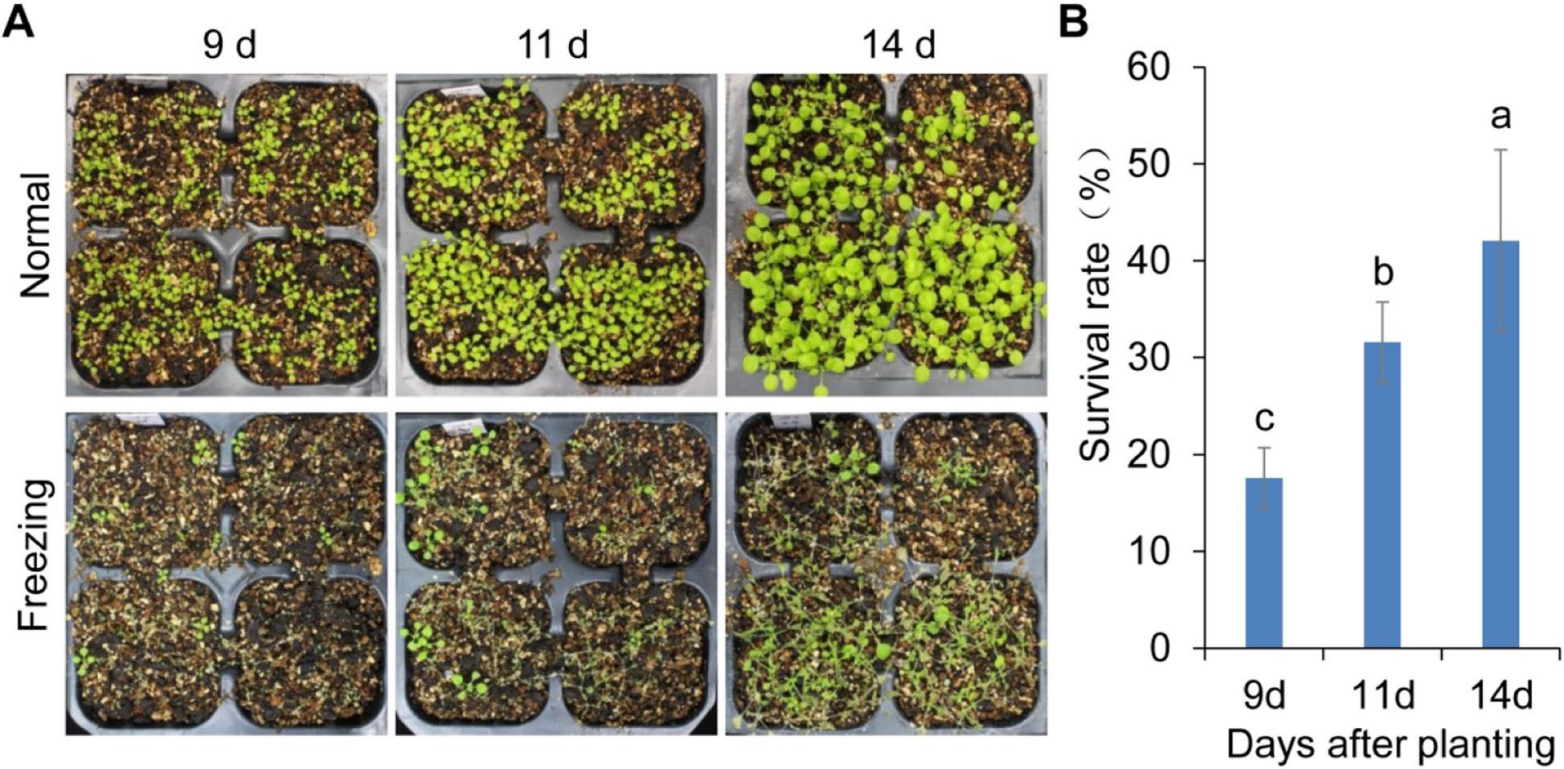
The analysis of freezing tolerance capacity at different vegetative phases. **A** Phenotypes of 9-day-old, 11-day-old and 14-day-old Col-0 plants under normal and freezing stress conditions. **B** Survival rate of Col-0 plants after cold-treatment. Statistically distinct genotypes were identified by one-way ANOVA with post hoc Least Significant Difference (LSD) multiple comparison test. The pairwise comparison results are presented by inserting letters to describe statistics information: different letters indicate significant difference between genotypes at *P* < 0.05

To understand the mechanistic connection between freezing tolerance and vegetative development, we first confirmed the growth time point of vegetative developmental stages and examined the capacity of freezing tolerance in different vegetative development stages under long-day condition. The Col-0 plants produced abaxial trichomes on leaf 5.2 at about 11 days after transferring to green house, suggesting that the 11^th^ -day was the developmental transition time (Fig. S1A, B) from juvenile phase to adult phase. Therefore, we chose the 9-day-old (as juvenile stage), 11-day-old (as transition stage) and 14-day-old (as adult stage) plants to perform the freezing-treatment experiment. After freezing-treatment, the bigger plants had more surviving ones (Fig. 1A). The statistical results showed that the survival rate of 9-day-old plants was 17.6%, which was significant lower than that of 11-day-old plants (31.6%); and the survival rate of 11-day-old plants was significant lower than that of 14-day-old plants (42.1%) (Fig. 1B). The result indicated that the capacity of freezing tolerance was increased with plant age, suggesting that the freezing-tolerance during vegetative stage was age-dependent.

### Overexpression of *SPL9* enhances the freezing tolerance

**Fig. 2:**
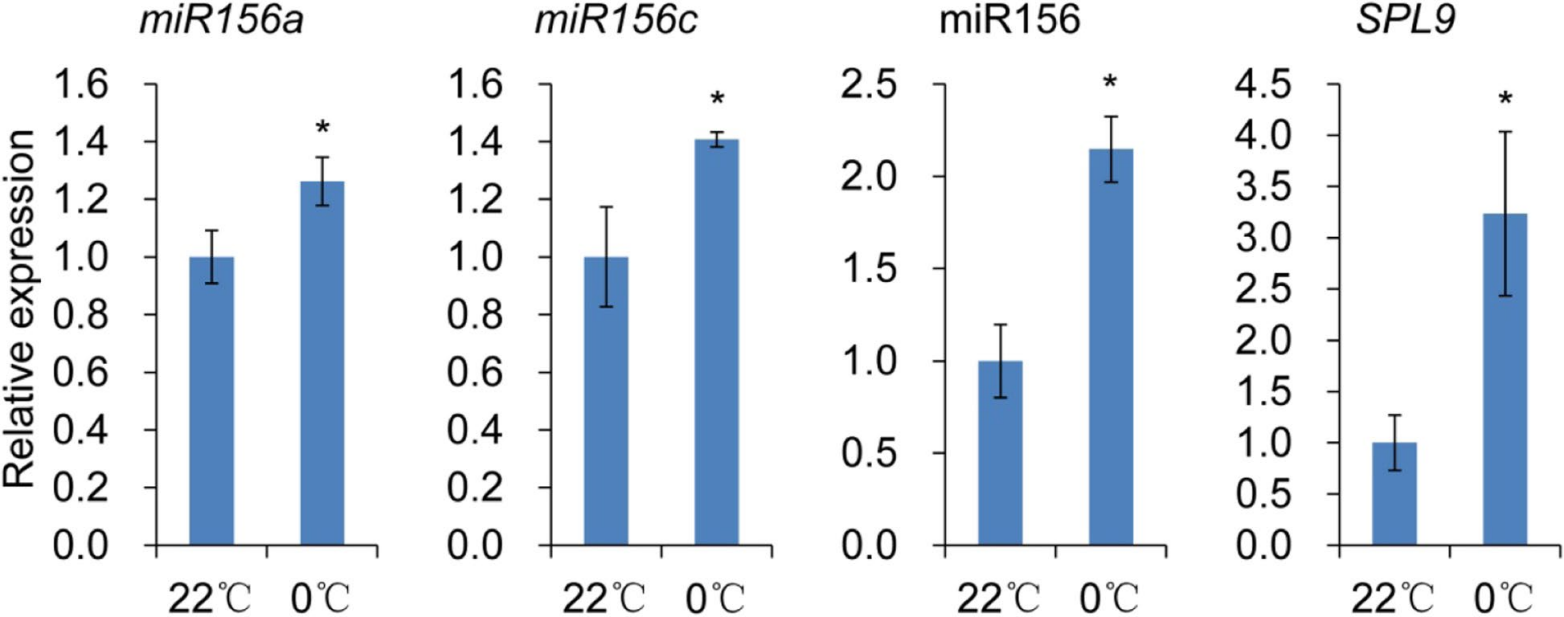
The expression of *miR156a*, *miR156c*, miR156 and *SPL9* under 22 °C and 0 °C by RT-qPCR. Asterisks indicate significant difference from 22 °C using Student’s *t*-test (* *P* < 0.05)

The conserved miR156-SPL pathway has been shown to regulate vegetative phase change and involve in cold response in plants [7, 51]. However, how miR156-SPL pathway responds to the low temperature remains unclear. We analyzed the expression of miR156-SPL pathway genes after low-temperature exposure and found that the levels of mature miR156 and its pri-mRNAs, *miR156a* and *miR156c*, were elevated under low temperature (Fig. 2). The expressions of *SPL3* and *SPL13*, targets of miR156, were correspondingly reduced (Fig. S2), whereas the expression of another target, *SPL9*, was upregulated under low temperature (Fig. 2), suggesting that SPL9 might be positively involved in cold response. However, herein low temperatures induced the expression of both miR156 and *SPL9*, inconsistent with the normal miR156-SPL regulation. We detected the expression of *SPL9* in Col-0, *156OE*, *MIM156* and *rSPL9* plants under normal and cold conditions. The results showed that low temperature induced the expression of *SPL9* in Col-0, *156OE*, and *MIM156* plants, whereas the level of *SPL9* was lower in *156OE* plants and higher in *MIM156* plants than in Col-0, indicating that *SPL9* was regulated by both miR156 and low temperature (Fig. S3). Interestingly, *SPL9* expression was also elevated in *rSPL9* plants, as miR156-insensitive *SPL9-*overexpressing transgenic plants, under low temperature condition (Fig. S3), showing that SPL9 might be partially regulated by low temperature via miR156-independent pathway.

**Fig. 3:**
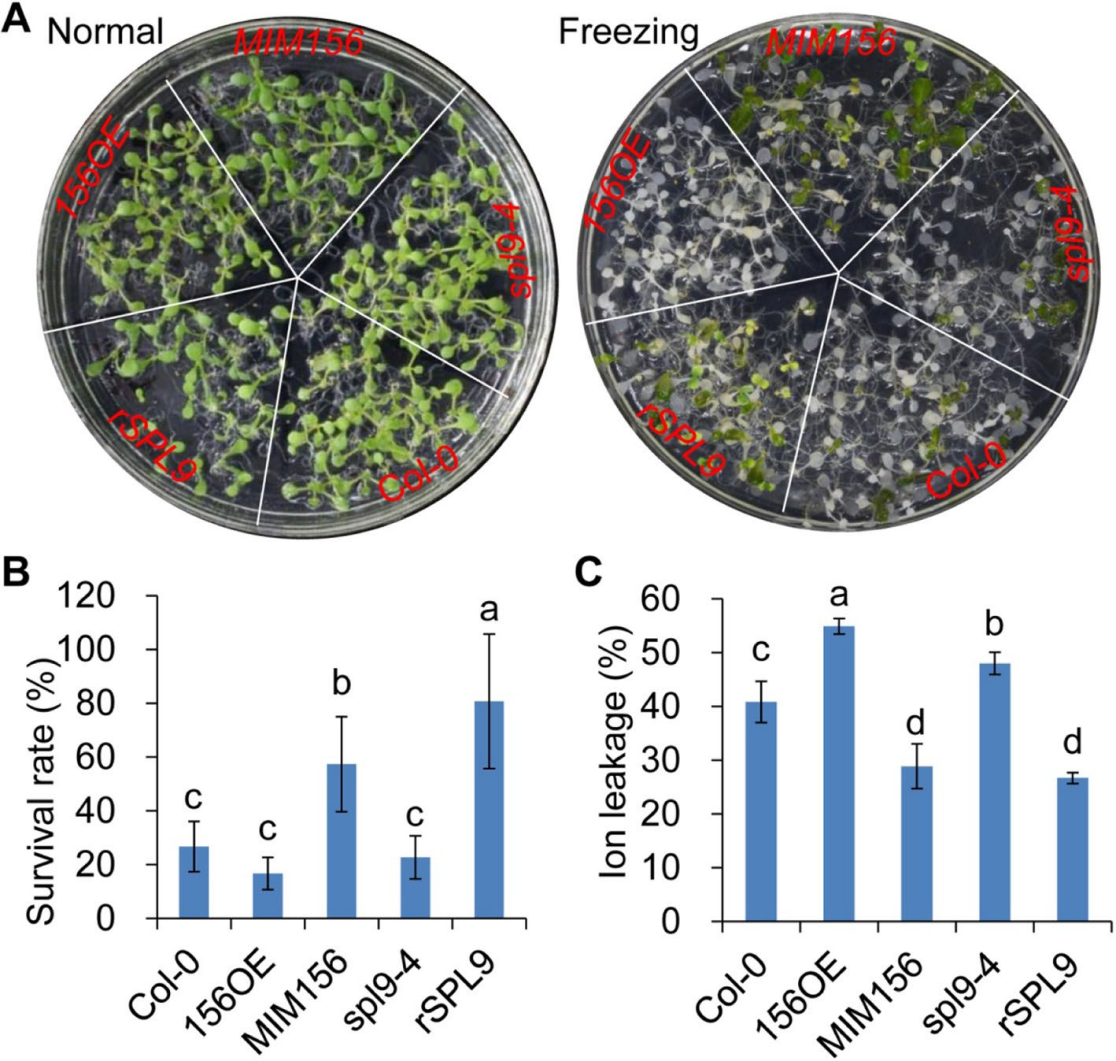
miR156-SPL pathway functions in freezing tolerance. **A** Phenotype of the Col-0, *35S:miR156a*, *UBI:MIM156*, *spl9–4* and *rSPL9* plants at normal and freezing stress conditions. **B** Survival rate of Col-0, *35S:miR156a*, *UBI:MIM156*, *spl9–4* and *rSPL9* plants after cold-treatment. Statistically distinct genotypes were identified by one-way ANOVA with post hoc LSD multiple comparison test. The pairwise comparison results are presented by inserting letters to describe statistics information: different letters indicate significant difference between genotypes at *P* < 0.05. **C** Ion leakage (%) of Col-0, *35S:miR156a*, *UBI:MIM156*, *spl9–4* and *rSPL9* plants after cold-treatment. Statistically distinct genotypes were identified by one-way ANOVA with post hoc LSD multiple comparison test. The pairwise comparison results are presented by inserting letters to describe statistics information: different letters indicate significant difference between genotypes at *P* < 0.05

To further examine the role of miR156-SPL9 pathway in freezing tolerance, we performed the freezing tolerance assay. The results showed that the *35S:miR156a* plants and the *spl9–4* mutant displayed reduced freezing tolerance based on the growth status, the survival rate and ion leakage as compared with Col-0 plants, whereas the *UBI:MIM156* plants and the *rSPL9* plants exhibited higher cold tolerance judged by their good growth status, the high survival rate and lower ion leakage than Col-0 plants (Fig. 3), as expected. This result indicated that overexpression of *SPL9* enhanced the plant freezing tolerance.

### SPL9 positively regulates the expression of *CBF2*

**Fig. 4:**
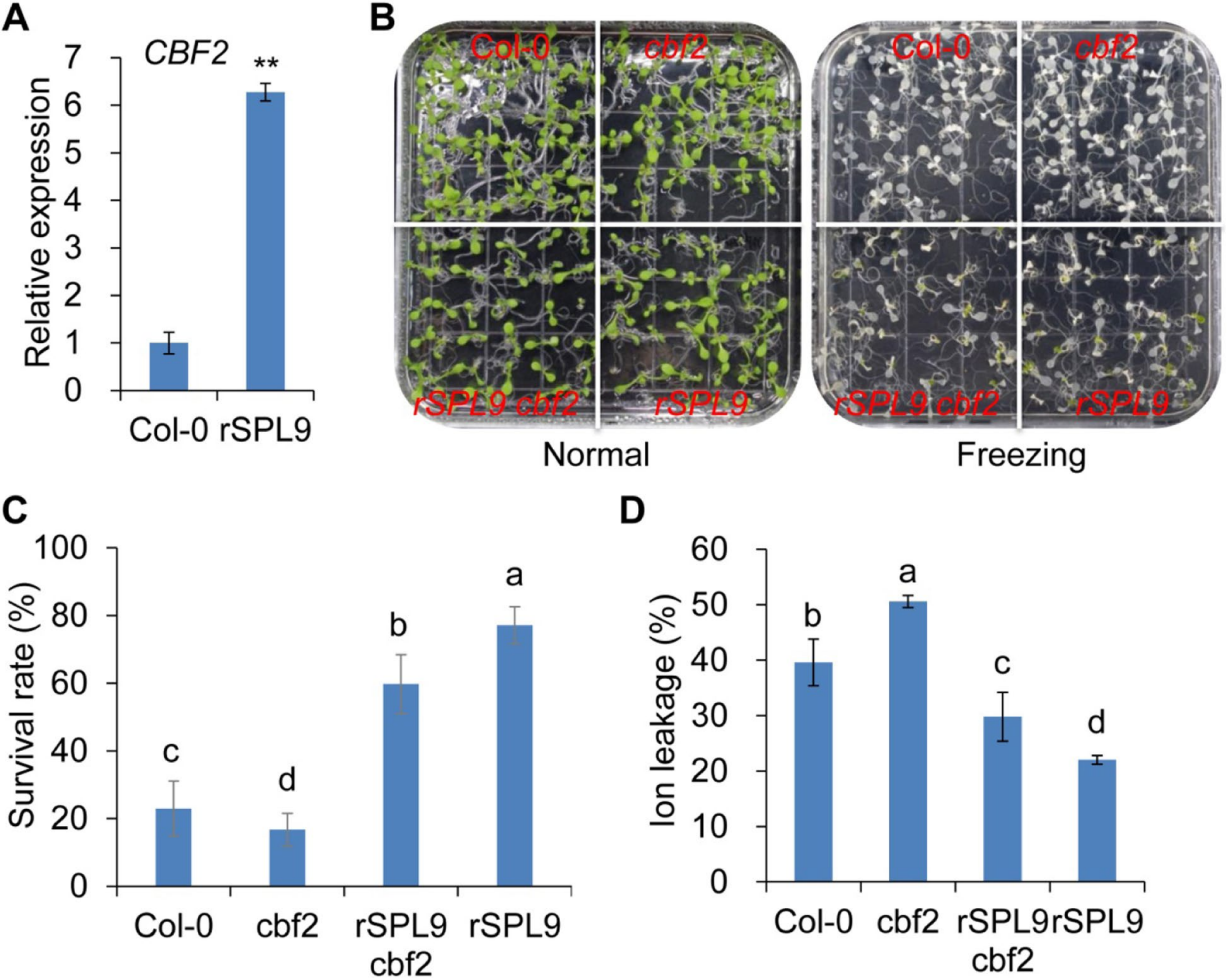
SPL9 acts upstream of *CBF2* on freezing tolerance. **A** The expression of *CBF2* in Col-0 and *rSPL9* plants by RT-qPCR. **B** Phenotype of the Col-0, *cbf2*, *rSPL9* and *rSPL9 cbf2* plants at normal and freezing stress conditions. **C** Survival rate of Col-0, *cbf2*, *rSPL9* and *rSPL9 cbf2* plants after freezing-treatment. Statistically distinct genotypes were identified by one-way ANOVA with post hoc LSD multiple comparison test. The pairwise comparison results are presented by inserting letters to describe statistics information: different letters indicate significant difference between genotypes at *P* < 0.05; the same letter indicates no significant difference between different genotypes. This experiment was biologically repeated for three times. **D** Ion leakage (%) of Col-0, *cbf2*, *rSPL9* and *rSPL9 cbf2* plants after freezing-treatment. Statistically distinct genotypes were identified by one-way ANOVA with post hoc LSD multiple comparison test. The pairwise comparison results are presented by inserting letters to describe statistics information: different letters indicate significant difference between genotypes at *P* < 0.05; the same letter indicates no significant difference between different genotypes. This experiment was biologically repeated for three times

CBF2 is the master factor in regulation of cold tolerance [54]. Overexpression of *CBF2* [54] and Overexpression of *SPL9* both enhanced the freezing tolerance in *Arabidopsis* (Fig. 3). However, whether SPL9 relays the downstream signal *CBF2* to mediate freezing tolerance is unknown. We first determined the expression of *CBF2* in *rSPL9* plants and Col-0, and the expression of *SPL9* in *cbf2* under normal condition using RT-qPCR. The expression of *CBF2* was induced more than 6-fold in *rSPL9* plants (Fig. 4A), but there was no significant change in the expression of *SPL9* in *cbf2* mutant (Fig. S4), indicating that SPL9 functions upstream of *CBF2* to activate *CBF2* expression. Under low temperature condition, *CBF2* expression was induced in Col-0, *spl9–4* and *rSPL9* plants, whereas the expression of *CBF2* in *rSPL9* plants was relative lower than that in Col-0 (Fig. S5). Next, we asked if the expression of affected genes acting downstream of cold signaling in *rSPL9* plants was also CBF2 dependent. We examined the expression of CBF2-regulated genes in Col-0 and *rSPL9* plants. The expression of *KINASE 1* (*KIN1*), *RESPONSIVE TO DESICCATION 29A* (*RD29A*), and *COR47* was all significantly up-regulated in *rSPL9* plants compared with Col-0 under normal condition; while the expression of *KIN1* was significantly up-regulated in *rSPL9* plants compared with Col-0 under cold condition and the expression of *RD29A*, and *COR47* was down-regulated in *rSPL9* plants compared with Col-0 under cold condition (Fig. S6). These findings do support the concept of developmental priming, which aims to protect developing *rSPL9* seedlings against cold stress by elevating expression of *CBF2*.

To investigate the genetic interaction between SPL9 and CBF2 in regulating freezing response, we crossed *cbf2* with *rSPL9* to generate the *rSPL9 cbf2* plants. The freezing-tolerance assay showed that *cbf2* plants were more sensitive to cold with lower survival rate and higher ion leakage than Col-0; *rSPL9 cbf2* plants exhibited lower survival rate and higher ion leakage than *rSPL9*, and exhibited greater survival rate and lower ion leakage than Col-0 and *cbf2* (Fig. 4B, C, D), indicating SPL9 partially contributes to CBF2-mediated freezing tolerance in plants. Moreover, we introduced the *rSPL9* to *pCBF2:GUS* plants to generate *pCBF2:GUS rSPL9* plants. The GUS activity of *pCBF2:GUS* plant was high in the shoot meristem, but significantly lower than that of *pCBF2:GUS rSPL9* plants (Fig. S7), demonstrating that SPL9 might enhance the activity of *CBF2* promoter. These results suggested that SPL9 positively regulated the expression of *CBF2* against freezing stress.

### SPL9 promotes the transcription of *CBF2* by directly binding to *CBF2* promoter

**Fig. 5:**
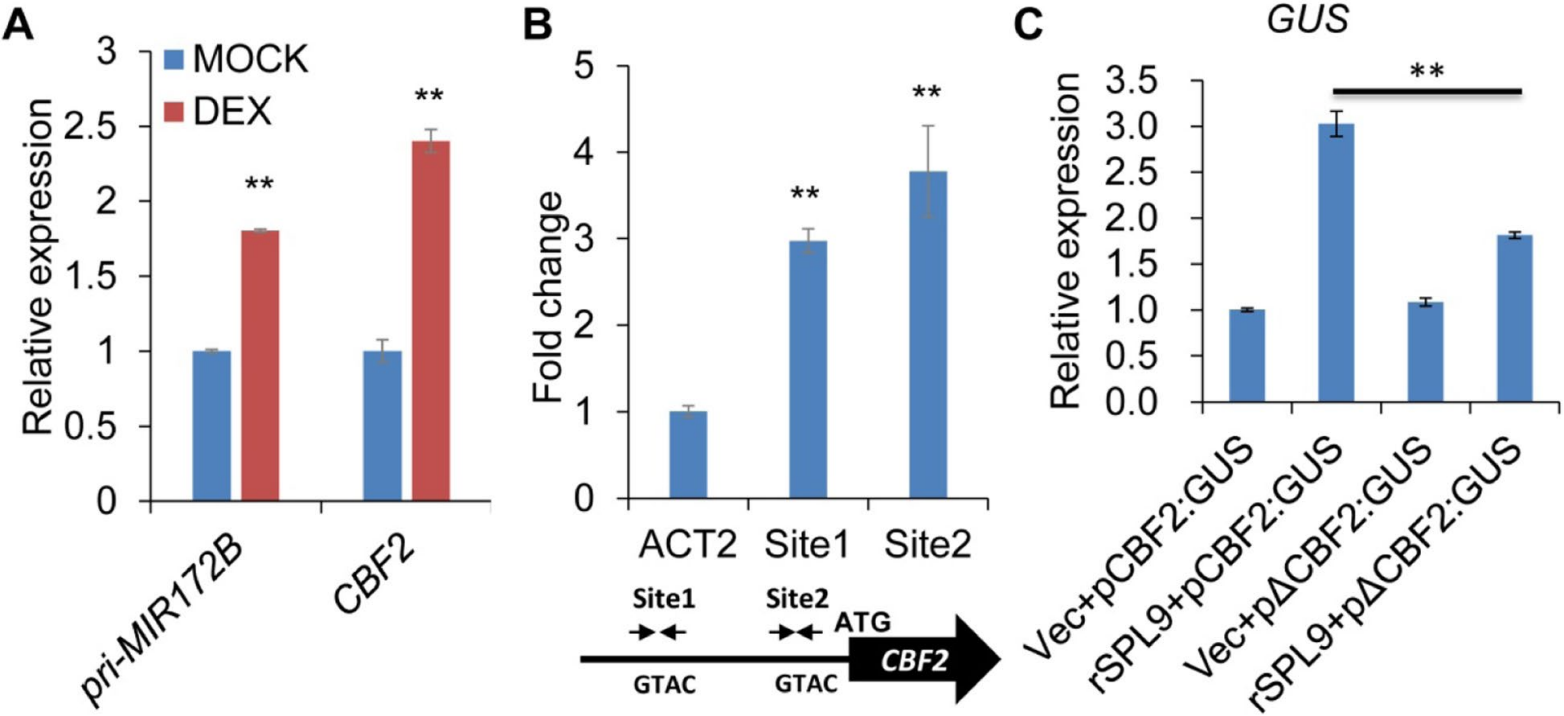
SPL9 directly promotes *CBF2* expression in *Arabidopsis*. **A** RT-qPCR analysis of *miR172B* and *CBF2* expression, respectively, after SPL9 activation in the presence of DEX. Three biological replicates were performed. Asterisks indicate significant difference from mock using Student’s *t*-test (*P* < 0.01). **B** ChIP-qPCR analysis of SPL9 binding sites in the promoter of *CBF2*. Chromatins from 9-day-old *pSPL9:3 × FLAG-rSPL9* and *rSPL9* (as negative control) seedlings grown in long day condition were immunoprecipitated with a polyclonal antibody to FLAG. Values are the means of three biological replicates, each having three technical replicates. Site1 and Site2 denote different PCR-amplified regions in the promoter region of *CBF2*. *ACT2* was a negative control. Asterisks indicate significant difference from the value of *ACT2* using Student’s *t*-test (*P* < 0.01). **C** Activation of *CBF2* transcription by direct binding of SPL9 to the *cis*-regulatory sequence in the promoter of *CBF2*. *GUS* expression was quantitated using RT-qPCR in leaves of *N. benthamiana* infiltrated with *Agrobacterium* with different combinations of constructs, including Vec + pCBF2:GUS, Vec + pΔCBF2:GUS, rSPL9 + pCBF2:GUS, and rSPL9 + pΔCBF2:GUS. pSY06 (Vec): the control vector. Values were normalized to that of Vec + pCBF2:GUS and are the mean of three biological replicates; Asterisks indicate significant difference from Vec + pCBF2:GUS using Student’s *t*-test (** *P* < 0.01)

To further investigate if *CBF2* is a direct transcriptional target of SPL9, we used an inducible glucocorticoid receptor (GR) expression system to test this. The result showed that the expression of *miR172B*, a positive control, was elevated about 1.8-fold in DEX-treated *pSPL9:GR-rSPL9* samples compared with mock; and the expression of *CBF2* was induced about 2.4-fold in DEX-treated *pSPL9:GR-rSPL9* samples compared with mock, this result demonstrated that *CBF2* is a direct transcriptional target of SPL9 (Fig. 5A). In addition, *CBF1* and *CBF3* are important to cold response in plants. Therefore, we determined whether SPL9 directly regulated the expression of *CBF1* and *CBF3* by GR system, whereas no significant changes of *CBF1* and *CBF3* expressions were found in mock and DEX-treated *pSPL9:GR-rSPL9* samples (Fig. S8), indicating that SPL9 did not directly regulate the expression of *CBF1* and *CBF3* in *Arabidopsis*.

It has been reported that SPL9 binds to the motif “GTAC” of target genes to regulate their expressions. Consequently, we detected two sites with “GTAC” in the *CBF2* promoter, and designed two pairs of primers surrounding those sites for ChIP-qPCR assay. The ChIP-qPCR analysis demonstrated that the DNA at the “Site1” and “Site2” was significantly enriched about 3.0-fold and 3.8-fold, respectively, compared with the negative control *ACTIN2* (*ACT2*) (Fig. 5B), implying that SPL9 regulates *CBF2* expression by directly binding to the promoter sequence of *CBF2* gene.

To test the significance of direct binding of *SPL9* to the *cis*-regulatory element in the promoter of *CBF2*, we constructed a *CBF2*-promoter-driven *GUS* vector pCBF2:GUS, and a mutated pΔCBF2:GUS vector with a mutated site “tgca” (the wild type is “GTAC”). The transient activation assay indicated that the expression of *GUS* and the GUS staining in the combination rSPL9 + pCBF2:GUS were higher than that in Vec + pCBF2:GUS (Fig. 5C, S9), suggesting that SPL9 activated the expression of *CBF2*. The expression of *GUS* and the GUS staining in the combination rSPL9 + pΔCBF2:GUS were also higher than that in Vec + pΔCBF2:GUS (Fig. 5C, S9). However, the *GUS* transcript level and the GUS staining in the combination rSPL9 + pΔCBF2:GUS were significantly lower than that in the combination rSPL9 + pCBF2:GUS (Fig. 5C, S9), indicating that the “GTAC” site was of great importance for the SPL9 binding to and regulating. These results suggested that SPL9 promotes the transcription of *CBF2* by directly binding to the “GTAC” site of *CBF2* promoter for the cold response.

## Discussion

Ontogenetic resistance against biotic and abiotic stresses has been extensively studied in plants [6, 15, 30, 43]. Abiotic stress tolerances, including salt, drought and heat stress tolerances are decreased with plant age, while we show here that the cold tolerance is increased with plant age. Cold stress has a profound impact on plant ontogenetic development, leading to growth repression and reduced yields. Overexpression of miR156 or loss-of-function of *SPL9* delays the ontogenetic development, induces the drought, heat and salt tolerances, and decreases the cold tolerance in *Arabidopsis* and rice [6, 7, 43]. To conclude, it is convinced that miR156 served opposite roles in respond to cold stress and salt/drought/heat stresses. And it is still a mystery what mechanism drives the differential responses of miR156-SPL pathway. In this study, we found a molecular link between SPL9 and CBF2, the master transcription regulators in age- and cold-signaling pathways, respectively. SPL9 directly binds to *CBF2* promoter to activate *CBF2* expression as developmental priming for the protection of shoot development under unexpected cold stress.

To date, the CBF-dependent cold signaling pathway has been studied widely. CBF1/2/3, APETALA2/ETHYLENE-RESPONSIVE (AP2/ERF1) family transcription factors, directly bind to the conserved CRT/DRE motifs of *COR* genes to activate their expression for cold response [18, 33, 34, 44, 47]. Overexpressing *CBFs* increases the expression of *COR* genes and enhances freezing tolerance in plants [46]. Consequently, some factors, positively or negatively regulating the *CBFs* expression, act critical roles in the cold response. ICE1 directly binds to *CBF2* promoters to activate the expression of *CBF* genes for increasing freezing tolerance [29]. Mitogen-activated protein kinase 3 (MPK3) and MPK6 interact with and phosphorylate ICE1, hereafter reduces its stability and transcriptional activity, and finally represses the *CBFs* and *CORs* transcripts [31] and freezing tolerance. AtMYB15 and OsMYBS3 directly repress the expression of *CBFs* and negatively regulate chilling tolerance in *Arabidopsis* and rice, respectively [2, 8]. In this study, we found another transcription factor SPL9 directly bound to the “GTAC” motif of *CBF2* promoter to active *CBF2* expression and thus enhanced the freezing tolerance against unexpected freezing stress.

It is of exceeding importance that the timing of the developmental stage transition determines the success of reproduction. SPL9 acts in regulation of vegetative phase change and floral transition by promoting the expression of *miR172B* and *SOC1*, *FRUITFULL* (*FUL*) genes [48, 51]. Low temperature inhibits the plant development leading to late flowering by elevating the expression of miR156 and some flowering-related genes [28]. Herein, we assured that SPL9 was a key factor in regulation of ontogenetic development and freezing response. Loss-of-function of *SPL9* accelerates the leaf emergence, while *rSPL9* plants reduce the initial rate of leaves [51]. The plants with the changes of *CBFs* levels have the similar phenomenon in the ontogenetic development as SPL9-related plants. The *CBF*-overexpressing plants display growth retardation and reduced plant biomass [1, 17, 23], whereas *cbf* triple mutants are larger than control under chilling stress [24]. Moreover, *SPL9* and *CBF2* are highly co-expressed in the shoot meristem. Therefore, SPL9 and CBF2 are considered as the important regulators for balancing plant growth and cold responses. The high levels of *CBF2* and CBF2 targets, *KIN1*, *RD29A* and *COR47*, in *rSPL9* plants under normal condition trigger the cold-defense priming. When the freezing stress occurs, the *rSPL9* plants can reduce stress response for less deleterious effect, while the wild type plants dramatically elevated the *CBF2* level to against cold injury by stopping development. Previous studies have shown that CBF2 is mainly involved in the regulation of cold acclimation [29, 36, 40, 54]. Herein, we found that the freezing-tolerance capability of *rSPL9* was no significant difference compared with Col-0 (Fig. S10) after cold acclimation, even though *CBF2* was activated by SPL9 in *Arabidopsis*. It’s possible that pre-cold treatment resulted in higher *CBF2* expression of Col-0 and *rSPL9* plants (Fig. S5), as well as reduced the difference of freezing tolerance between Col-0 and *rSPL9* plants. These results support our conclusion that the high expression of *CBF2* in the *rSPL9* plants under normal condition is critical for triggering cold defense priming against unexpected freezing stress.

As important direct target of SPL9, the expression of *CBF2* was significantly increased in *rSPL9* plants. However, the genetic results showed that the *cbf2* mutation only slightly affected the freezing phenotype of the *rSPL9* plants, suggesting that SPL9 may also regulate other components to promote freezing tolerance. Recent study indicates that BZR1, positive regulator in freezing tolerance [32], physically interacts with SPL9 to regulate the vegetative phase change in *Arabidopsis* [49]. And BZR1 positively modulates plant freezing tolerance through CBF-dependent and CBF-independent pathways [32]. Herein, we found that SPL9 could regulate CBF target genes, *KIN1*, *RD29A* and *COR47*, and CBF-independent COR genes, *WRKY6*, *SENESCENCE-ASSOCIATED GENE 21* (*SAG21*) and *SOC1* (Fig. S6), to respond to freezing stress. *SOC1*, as a direct target of SPL9 and BZR1 [32, 48], functions negatively in regulating plant responses to cold stress and positively in regulation of flowering [39]. *WRKY6* directly regulated by BZR1 positively modulates freezing tolerance [32]. Thus, SPL9 might serve as a mediator of the crosstalk in cold and developmental signaling pathways. Our results indicate that SPL9, similar with BZR1, positively controls plant freezing tolerance via CBF-dependent and CBF-independent pathways.

In this study, we detected that miR156 was increased under low temperature treatment in *Arabidopsis*, the same as miR535 [45] and difference with *OsmiR156k* in rice [7]. Correspondingly, the expression of *SPL3* and *SPL13*, target of miR156, were downregulated in *Arabidopsis*, whereas the transcript of *SPL9* was elevated, indicating that SPL9 was mainly regulated by cold, and partially regulated by miR156. The expression of *SPL9* in *rSPL9* plants was induced by low temperature, suggesting that the expression of *SPL9* may be independent miR156 under cold condition. This fine-tuned regulation by miR156-SPL indicates its function in balancing plant growth and cold tolerance. Previous studies have demonstrated the roles of miR156 superfamily including miR156, miR529, and miR535 in repressing the cold tolerance in rice. In rice, four SPL genes including *OsSPL2*, *OsSPL14*, *OsSPL17* and *OsSPL18* are co-targeted by OsmiR156/miR529/miR535; and six SPL genes, including *OsSPL4*, *OsSPL7*, *OsSPL11*, *OsSPL12*, *OsSPL16* and *OsSPL19* are co-targeted by OsmiR156/miR535 [45]. Overexpression of *OsmiR156k*, which is down-regulated under cold stress, reduces tolerance to cold stress in rice by suppressing the expression of *Os01g22249*, *OsP5CS* (*Oryza sativa DELTA1-PYRROLINE-5-CARBOXYLATE SYNTHASE*), *OsSPL3*, *OsSPL14* and *OsSPL17* [7]. However, the expression of *OsmiR535* is rapidly induced by cold stress in rice. The *OsmiR535*-overpressing rice plants display significantly lower reactive oxygen species (ROS) scavenging enzyme activity and accumulate much more malondialdehyde (MDA) under cold stress than the wild type. Overexpression of *OsmiR535* inhibits the expression of *OsCBF1*, *OsCBF2*, and *OsCBF3* under normal and cold conditions and its targets, including *OsSPL14*, *OsSPL11* and *OsSPL4* [45]. But there is no evidence to certificate if SPL could directly bind to *CBFs* and regulate *CBFs* expression under cold stress in rice. These results indicate that *Arabidopsis* has different strategies to balance the cold stress and development compared with rice. In *Arabidopsis*, miR156 and *SPL9* are both induced by cold; miR156 functions in reducing the growth rate by repressing *SPL3* and *SPL13*, whereas its another target, SPL9, acts as a commander to direct the expression of *CBF2* against the cold damage by triggering defense priming. Most importantly, SPL9 functions in regulation of flowering [48]. If only the expression of *SPL9* is induced under cold condition, the plants will bloom earlier resulting in damage of floral organ caused by cold; while with the high level of miR156 under cold condition, the developmental transition of plants will be restrained to reduce the injury by low temperature. That’s a fine regulatory mechanism in balancing development and freezing stress in *Arabidopsis*. However, the rice miR156 and miR535 have adverse expression pattern under cold stress; the balance between miR156 and miR535 is critical for the interaction between cold and development. That’s to say different plants generate different mechanisms to respond to the freezing stress and maintain their lives by adjusting their growth conditions under harsh environments.

## Conclusions

**Fig. 6:**
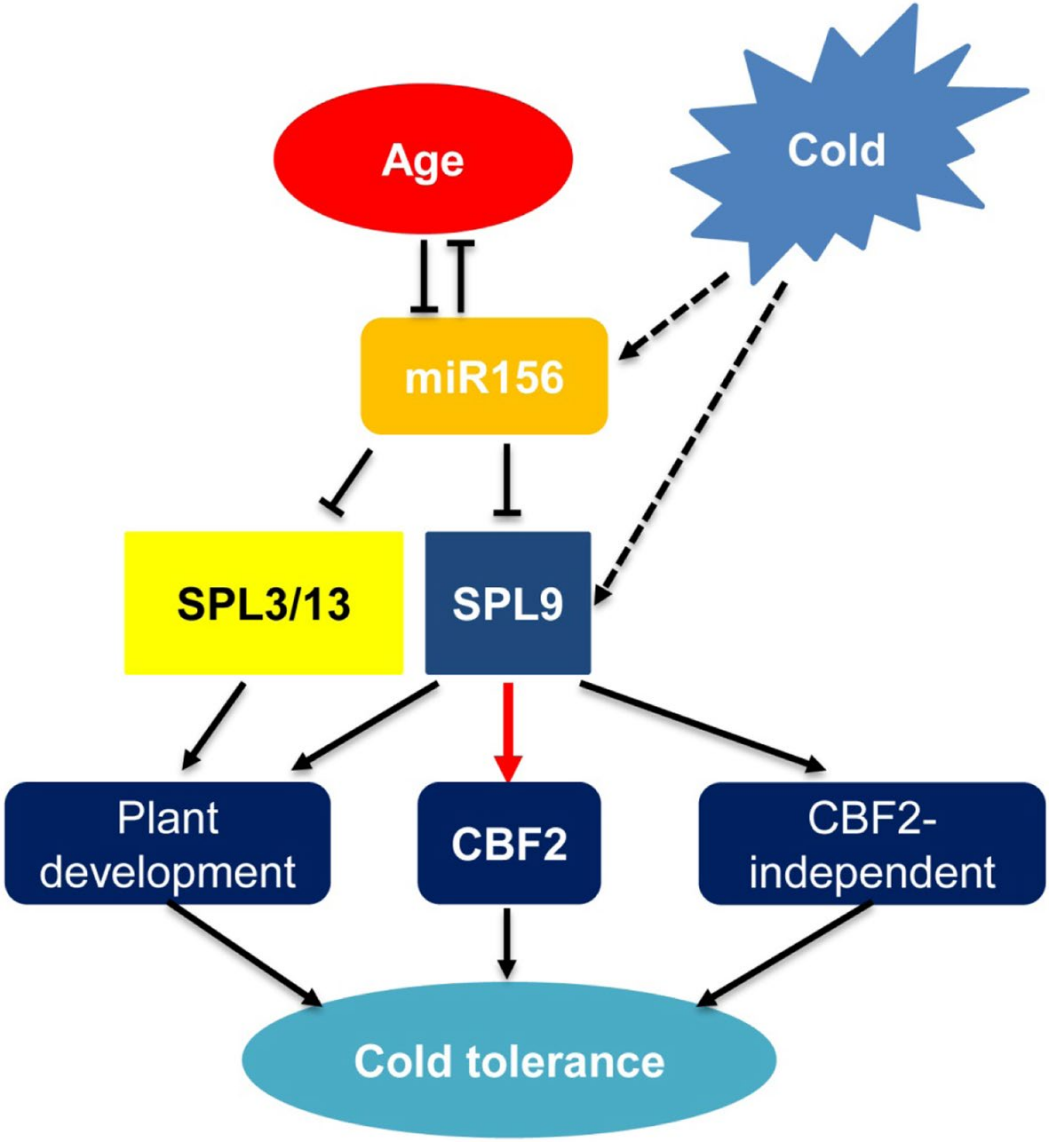
Model for SPL9-CBF2 complex in regulating freezing tolerance in *Arabidopsis*

Based on our findings, we propose a model for the role of SPL9 in regulation of freezing tolerance during the vegetative stage (Fig. 6). According to this model, age and low temperature induce the transcript of *SPL9*; the elevated SPL9 promotes the binding of *CBF2* promoter to facilities *CBF2* expression, and thus increases higher capacity to tolerate freezing. miR156, as a master age-regulator, is induced by cold, and then inhibits the expression of *SPL3* and *SPL13* to control the plant developmental transition. Moreover, the capacity of freezing tolerance is increased with plant age. Therefore, miR156-SPL pathway functions as a balance sponge to maintain the plant development and freezing tolerance. So far, crop improvement for cold tolerance through conventional breeding approach showed little success due to the complexity of stress tolerance traits. This work promotes this process, deciphers the mechanisms underlying the response to cold stress and is of significant importance for boosting the breeding of cold-tolerant plant varieties.

## Materials and methods

### Plant material and growth conditions

The plant materials, including *35S:miR156a* (*156OE*, miR156 overexpressing line), *UBI:MIM156* (*MIM156*, miR156 repressing line by target mimic), *spl9–4* (cs807258, null allele of *SPL9*), *pEG302a-rSPL9* (*rSPL9*, *SPL9*-promoter-driven miR156-insensitive *SPL9-*overexpressing line), *pSPL9:GR-rSPL9* (miR156-insensitive SPL9 protein fused with *Glucocorticoid Receptor* (*GR*) gene), and *pSPL9:3 × FLAG-rSPL9* (miR156-insensitive SPL9 protein tagged with 3 *×* FLAG), were in a Columbia-0 (Col-0) background as reported previously [51]. The *cbf2* (SALK_025195, Col-0 background) mutant was a kind gift from Dr. Juan Lin (College of Life Science, Fudan University, Shanghai, China). The *rSPL9 cbf2* plant was generated by crossing *rSPL9* with *cbf2* mutant. Seeds were sown in the media with soil and vermiculite (1:1), treated at 4 °C for 2 days, and then transferred to the long-day greenhouse (22 °C, 16-h light/8-h dark cycles, with light intensity 130 μmmol•m^− 2^•s^− 1^). The plant age, abaxial trichomes and leaf shapes were measured based on our previous report [19, 20].

### Freezing-treatment assay

All seeds used for freezing-treatment experiments were collected at the same time and were naturally dried at the room temperature. The 9-day-old (juvenile stage), 11-day-old (transition stage) and 14-day-old (adult stage) Col-0 plants grown in soil under long day condition were treated at − 11 °C for 5 h to analyze the age-dependent cold-tolerance. For freezing-treatment assay, seeds of each genotype were washed twice with sterile water, sterilized with 75% ethanol for 10 min, washed more than five times with sterile water, and put on the sterile paper until dried. The sterilized seeds were sown one by one on 1/2MS medium plates (1% sucrose, 1.5% agar, pH 5.8), treated at 4 °C for 2 days, and then transferred to the long-day growth chamber. Arabidopsis seedlings were grown at 22 °C under long day condition for 9 day, and then treated at − 11 °C for 5 h. Thereafter, the seedlings were treated at 4 °C for 1 h and were transferred to long day conditions for 5 days for recovery. The pictures were photographed, the surviving seedlings were counted and the survival rates (surviving seedling/total seedling× 100%) were measured. For the electrolyte leakage assay [22], the cold-treated seedlings were transferred carefully to tubes containing 10 mL of deionized water and vacuumed for 1 min, and the conductivity (S_0_) of the solution was measured. After 2-h treatment at room temperature, the conductivity (S_1_) of the solution was measured again. Next, the tubes with the samples were boiled for 5 min. After cooling down to room temperature, the boiled conductivity (S_2_) of the solution was measured finally. The percentage of electrolyte leakage was calculated: ion leakage (%) = (S_1_-S_0_) / (S_2_-S_0_) × 100%. For the cold-acclimation assay, the plants were treated at 4 °C for 3 days, and then treated at − 11 °C for 6 h and recovered for 5 days. After that the survival rates were measured. The experiments had been done more than three biological repeats under the same conditions in this study.

### RNA extraction and real-time quantitative PCR (RT-qPCR)

The 9-day-old seedlings were treated at 0 °C for 3 h, and then were sampled. RNA extraction, first-strand cDNAs synthesis, and RT-qPCR of mRNA and miRNA were carried out as previous report [19]. The Arabidopsis *TUBULIN BETA CHAIN 2* (*TUB2*) gene was as internal control for mRNA detection, and SNOR101 was as internal control for miRNA detection, respectively. The primers were listed in Table S1. All experiments had been done more than three times.

### GR induction assay

The 9-day-old *pSPL9:GR-rSPL9* seedlings were harvested and immersed in 0.1% ethanol (as mock), or 10 mM Dexamethasone (DEX) in 0.1% ethanol in 50-mL tubes. Thereafter, the treated samples were put into the long-day growth chamber to incubate for 8 h, and then were used to extract total RNAs for the expression analysis of *CBF2, CBF1, CBF3* and *miR172B* (as positive control) by RT-qPCR.

### Chromatin immunoprecipitation quantitative PCR (ChIP-qPCR) analysis

The SPL9 binding site, “GTAC” sequence, in the promoter of *CBF2* was analyzed. The ChIP-qPCR primers (Table S1) were designed based on the predicted SPL9-binding sites. About three grams of 9-day-old *pSPL9:3 × FLAG-rSPL9* and *rSPL9* (as a negative control) seedlings under long-day condition were harvested. ChIP-qPCR was performed as previous report [19]. This experiment had more than three biological replicas.

### Transient activation assay

The about 1-kb promoter of *CBF2* genes were amplified and fused into the pCAMBIA3301 vector to generate pCBF2:GUS vector. The *CBF2 cis*-regulatory sequences (GTAC) possibly bound to by SPL9 were mutated to “tgca” using overlapping PCR to generate pΔCBF2:GUS vector. The control vector (pSY06, Vec), UBI:rSPL9 (rSPL9), pCBF2:GUS and pΔCBF2:GUS were transformed into *Agrobacterium tumefaciens* strain GV3101, respectively; and then they were grouped into four combinations (Vec + pCBF2:GUS, Vec + pΔCBF2:GUS, rSPL9 + pCBF2:GUS, and rSPL9 + pΔCBF2:GUS) by pairwise mixing (1:1). Transient activation assay was performed as previous report [19]. The *pCBF2:GUS* plants were crossed with *rSPL9* plants to generate *pCBF2:GUS rSPL9* plants. Moreover, the injected tobacco leaves were stained with GUS staining solution, decolorized with 75% alcohol, and photographed. The *pCBF2:GUS* transgenic Arabidopsis lines and *pCBF2:GUS rSPL9* Arabidopsis plants were examined for GUS activity by histochemical staining using previous experimental procedure [19].

### Statistical analysis

Statistically significant differences were analyzed using the SPSS software by *t*-test or one-way ANOVA with post hoc Least Significant Difference (LSD) multiple comparison test.

## Supplementary Information

**Additional file 1: Figure S1.** Leaf shape, abaxial trichome phenotypes, and leaf initiation rate of Col-0. (A) Twenty-one-day-old Col-0 plant grown in long days, and its leaf shape and abaxial trichome phenotypes. Numbers indicate the first leaf with abaxial trichomes. (B) Leaf initiation rate of Col-0 in long days. Leaf numbers were scored at 7, 9, 11, 13, and 15 days (d) after transferring to greenhouse. The intersection point of two red lines represents the time and the leaf position when the vegetative phase transition is occurred. **Figure S2.** The expression of *SPL3* and *SPL13* in Col-0 under 22 °C and 0 °C by RT-qPCR. Asterisks indicate significant difference from 22 °C using Student’s *t*-test (** *P* < 0.01). **Figure S3.** The expression of *SPL9* in Col-0, *156OE*, *MIM156* and *rSPL9* plants under 22 °C and 0 °C by RT-qPCR. Asterisks indicate significant difference from 22 °C using Student’s *t*-test (** *P* < 0.01). **Figure S4.** The expression of *SPL9* by RT-qPCR in Col-0 and *cbf2* plants. ns, not significant. **Figure S5.** The expression of *CBF2* in Col-0, *spl9–4* and *rSPL9* plants under 22 °C and 0 °C by RT-qPCR. The numbers indicate the fold change of relative expression level. **Figure S6.** Expression of *KIN1*, *RD29A, COR47*, *WRKY6*, *SAG21*, and *SOC1*, genes in Col-0 and *rSPL9* plants under 22 °C and 0 °C by RT-qPCR. The expression levels of the genes in Col-0 at 22 °C were set as 1.0. Data are mean ± SD of two biological replicates. **Figure S7.** GUS activity by histochemical staining in *pCBF2:GUS* and *rSPL9 pCBF2:GUS* plants*.***Figure S8.** RT-qPCR analysis of *CBF1* and *CBF3* expression, respectively, after SPL9 activation in the presence of DEX. ns, not significant. **Figure S9.** GUS activity by histochemical staining in leaves of *N. benthamiana* infiltrated with *Agrobacterium* with different combinations of constructs, including Vec + pCBF2:GUS, Vec + pΔCBF2:GUS, rSPL9 + pCBF2:GUS, and rSPL9 + pΔCBF2:GUS. **Figure S10.** Freezing phenotypes and survival rates of Col-0 and *rSPL9* plants after cold-acclimation treatment. ns, not significant. **Table S1.** Primers used in this study.

## Data Availability

The data and materials in the current study are available from the corresponding author on reasonable request.

## Abbreviations

miR156: microRNA156
SPL9: SQUAMOSA PROMOTER BINDING PROTEIN-LIKE 9
CBF2: C-REPEAT BINDING FACTOR 2
DFR: DIHYDROFLAVONOL-4-REDUCTASE
IPA1: IDEAL PLANT ARCHITECTURE1
FT: FLOWERING LOCUS T
SOC1: SUPPRESSOR OF OVEREXPRESSION OF CO 1
ICE1: INDUCER OF CBF EXPRESSION 1
CBF/DREB1: C-REPEAT BINDING FACTOR/DRE BINDING FACTOR1
COR: Cold-Regulated genes
ZAT12: ZINC FINGER OF ARABIDOPSIS THALIANA 12
CAMTA: CALMODULIN-BINDING TRANSCRIPTION ACTIVATORS
PIF: PHYTOCHROME-ASSOCIATED PROTEIN
CES: CESTA
EIN3: ETHYLENE-INSENSITIVE 3
CCA1: CIRCADIAN CLOCK-ASSOCIATED 1
RVE4/LCL1: REVEILLE4/LHY-CCA1-Like 1
OST1: OPEN STOMATA 1
BIN2: BRASSINOSTEROID-INSENSITIVE2
BTF3: BASIC TRANSCRIPTION FACTOR 3
HOS1: HIGH EXPRESSION OF OSMOTICALLY RESPONSIVE GENE 1
BZR1: BRASSINAZOLE-RESISTANT 1
BES1: BRI1-EMS-SUPPRESSOR 1
LHY: LATE ELONGATED HYPOCOTYL
KIN1: KINASE 1
RD29A: RESPONSIVE TO DESICCATION 29A
ACT2: ACTIN2
AP2/ERF1: APETALA2/ETHYLENE-RESPONSIVE
FUL: FRUITFULL
SAG21: SENESCENCE-ASSOCIATED GENE 21
OsP5CS: *Oryza sativa* DELTA1-PYRROLINE-5-CARBOXYLATE SYNTHASE
ROS: Reactive oxygen species
MDA: Malondialdehyde
DEX: Dexamethasone
ChIP-qPCR: Chromatin immunoprecipitation quantitative PCR
LSD: Least Significant Difference
GR: Glucocorticoid receptor
TUB2: TUBULIN BETA CHAIN 2
