# Supplementary material for: SPL9 mediates freezing tolerance by directly regulating the expression of CBF2 in Arabidopsis thaliana
Source: BMC Plant Biol. 2022 Feb 2;22:59. doi: 10.1186/s12870-022-03445-8 (PMC8809014; doi:10.1186/s12870-022-03445-8)
Supplement: Supplementary file 1 — Additional file 1: Figure S1. Leaf shape, abaxial trichome phenotypes, and leaf initiation rate of Col-0. (A) Twenty-one-day-old Col-0 plant grown in long days, and its leaf shape and abaxial trichome phenotypes. Numbers indicate the first leaf with abaxial trichomes. (B) Leaf initiation rate of Col-0 in long days. Leaf numbers were scored at 7, 9, 11, 13, and 15 days (d) after transferring to greenhouse. The intersection point of two red lines represents the time and the leaf position when the vegetative phase transition is occurred. Figure S2. The expression of SPL3 and SPL13 in Col-0 under 22 °C and 0 °C by RT-qPCR. Asterisks indicate significant difference from 22 °C using Student’s t-test (** P < 0.01). Figure S3. The expression of SPL9 in Col-0, 156OE, MIM156 and rSPL9 plants under 22 °C and 0 °C by RT-qPCR. Asterisks indicate significant difference from 22 °C using Student’s t-test (** P < 0.01). Figure S4. The expression of SPL9 by RT-qPCR in Col-0 and cbf2 plants. ns, not significant. Figure S5. The expression of CBF2 in Col-0, spl9–4 and rSPL9 plants under 22 °C and 0 °C by RT-qPCR. The numbers indicate the fold change of relative expression level. Figure S6. Expression of KIN1, RD29A, COR47, WRKY6, SAG21, and SOC1, genes in Col-0 and rSPL9 plants under 22 °C and 0 °C by RT-qPCR. The expression levels of the genes in Col-0 at 22 °C were set as 1.0. Data are mean ± SD of two biological replicates. Figure S7. GUS activity by histochemical staining in pCBF2:GUS and rSPL9 pCBF2:GUS plants.Figure S8. RT-qPCR analysis of CBF1 and CBF3 expression, respectively, after SPL9 activation in the presence of DEX. ns, not significant. Figure S9. GUS activity by histochemical staining in leaves of N. benthamiana infiltrated with Agrobacterium with different combinations of constructs, including Vec + pCBF2:GUS, Vec + pΔCBF2:GUS, rSPL9 + pCBF2:GUS, and rSPL9 + pΔCBF2:GUS. Figure S10. Freezing phenotypes and survival rates of Col-0 and rSPL9 plants after cold-acclimation tr [file 12870_2022_3445_MOESM1_ESM.docx]

**Supplementary Material**

**
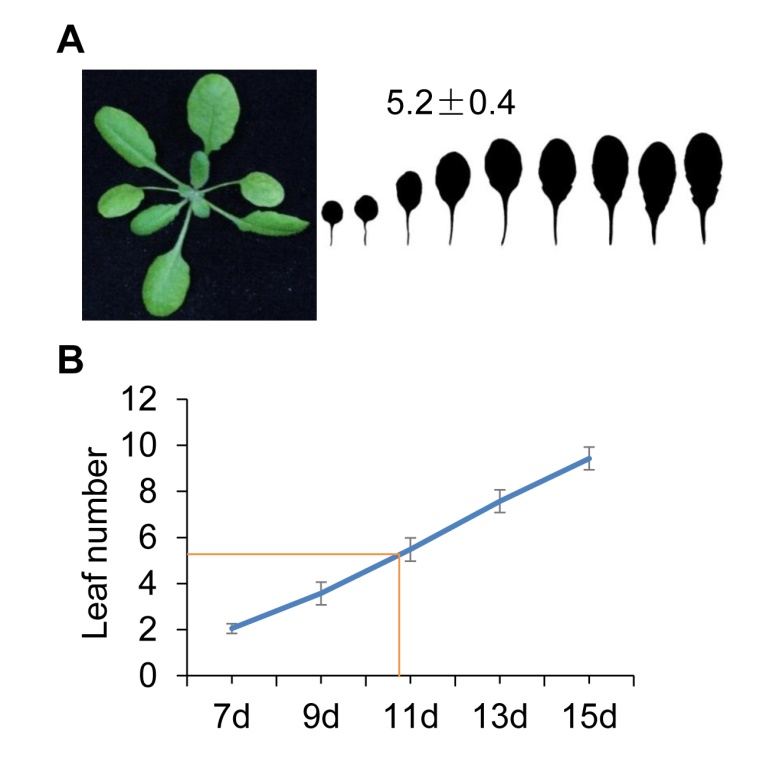
**

**Figure S1** Leaf shape, abaxial trichome phenotypes, and leaf initiation rate of Col-0.

(A) Twenty-one-day-old Col-0 plant grown in long days, and its leaf shape and abaxial trichome phenotypes. Numbers indicate the first leaf with abaxial trichomes.

(B) Leaf initiation rate of Col-0 in long days. Leaf numbers were scored at 7, 9, 11, 13, and 15 days (d) after transferring to greenhouse. The intersection point of two red lines represents the time and the leaf position when the vegetative phase transition is occurred.

**
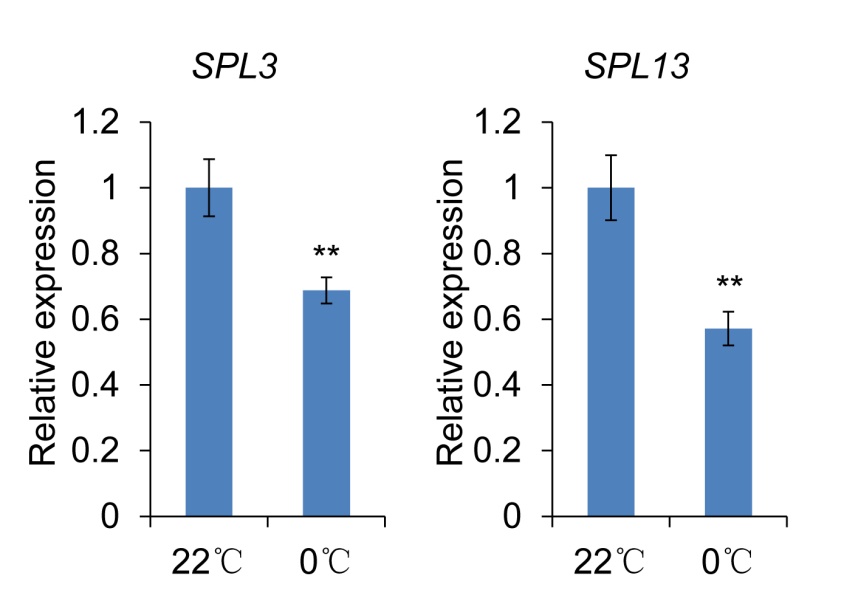
**

**Figure S2** The expression of *SPL3* and *SPL13* under 22℃ and 0℃ by RT-qPCR. Asterisks indicate significant difference from 22℃ using Student’s *t*-test (** *P* < 0.01).

**
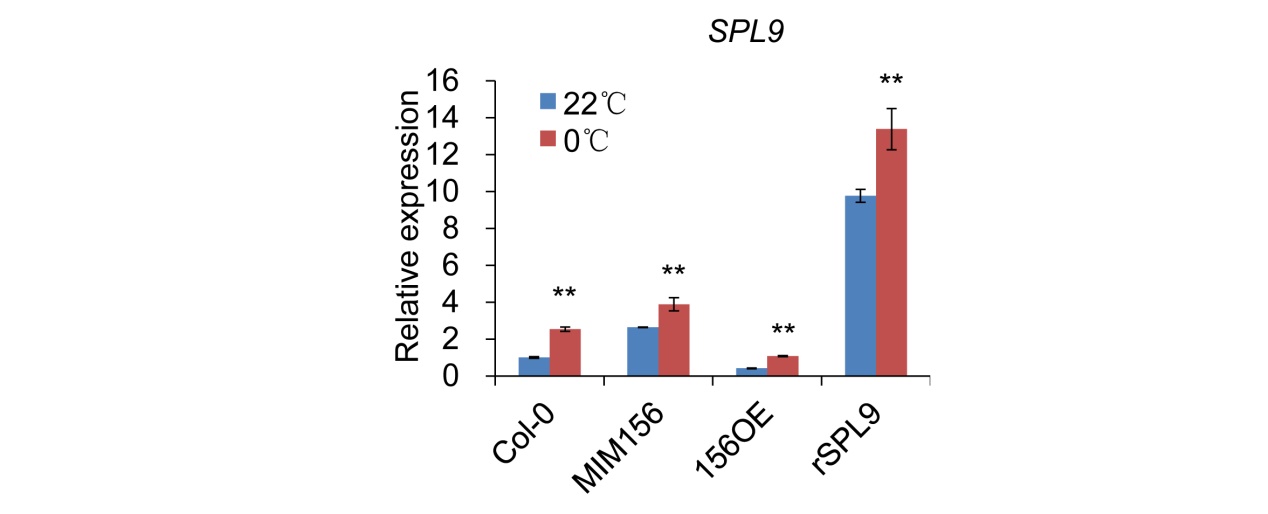
**

**Figure S3** The expression of *SPL9* in Col-0, *156OE*, *MIM156* and *rSPL9* plants under 22℃ and 0℃ by RT-qPCR. Asterisks indicate significant difference from 22℃ using Student’s *t*-test (** *P* < 0.01).

**
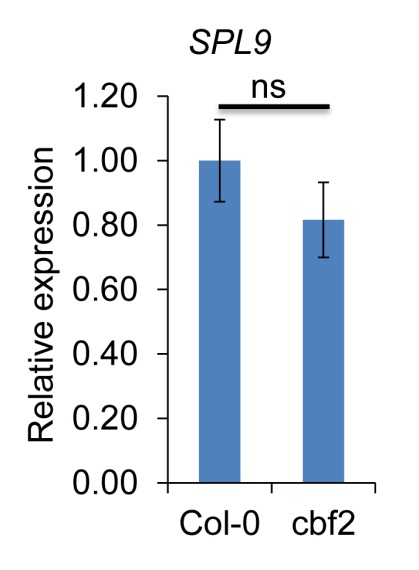
**

**Figure S4** The expression of *SPL9* by RT-qPCR in Col-0 and *cbf2* plants. ns, not significant.

**
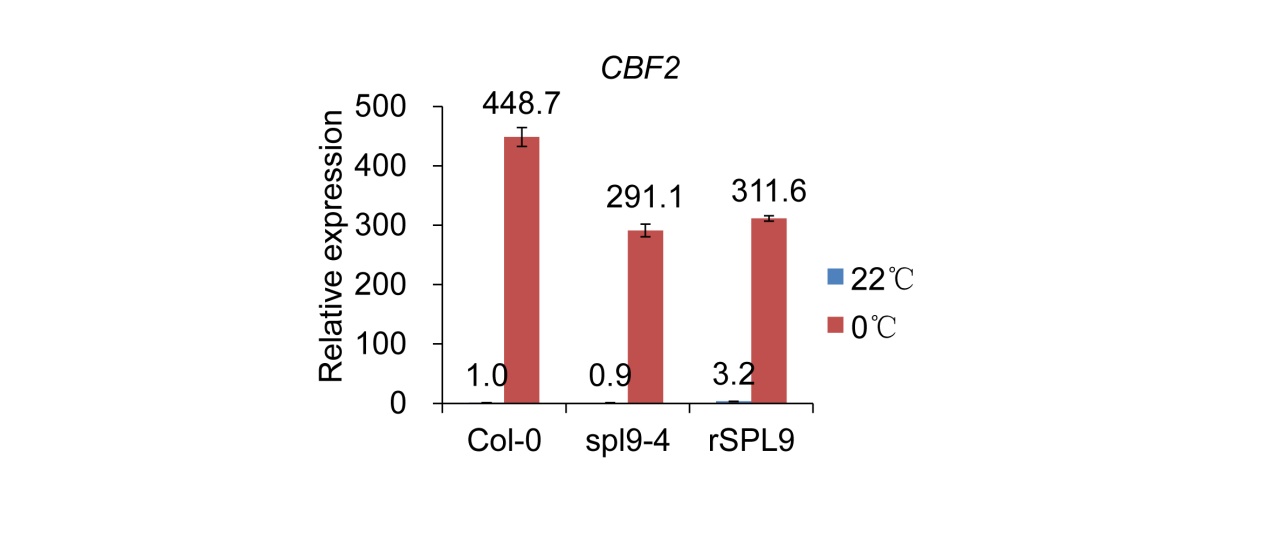
**

**Figure S5** The expression of *CBF2* in Col-0, *spl9-4* and *rSPL9* plants under 22℃ and 0℃ by RT-qPCR. The numbers indicate the fold change of relative expression level.

**Figure S6** Expression of *KIN1*, *RD29A,* *COR47*, *WRKY6*, *SAG21*, and *SOC1*, genes in Col-0 and *rSPL9* plants under 22℃ and 0℃ by RT-qPCR. The expression levels of the genes in Col-0 at 22℃ were set as 1.0. Data are mean ± SD of two biological replicates.


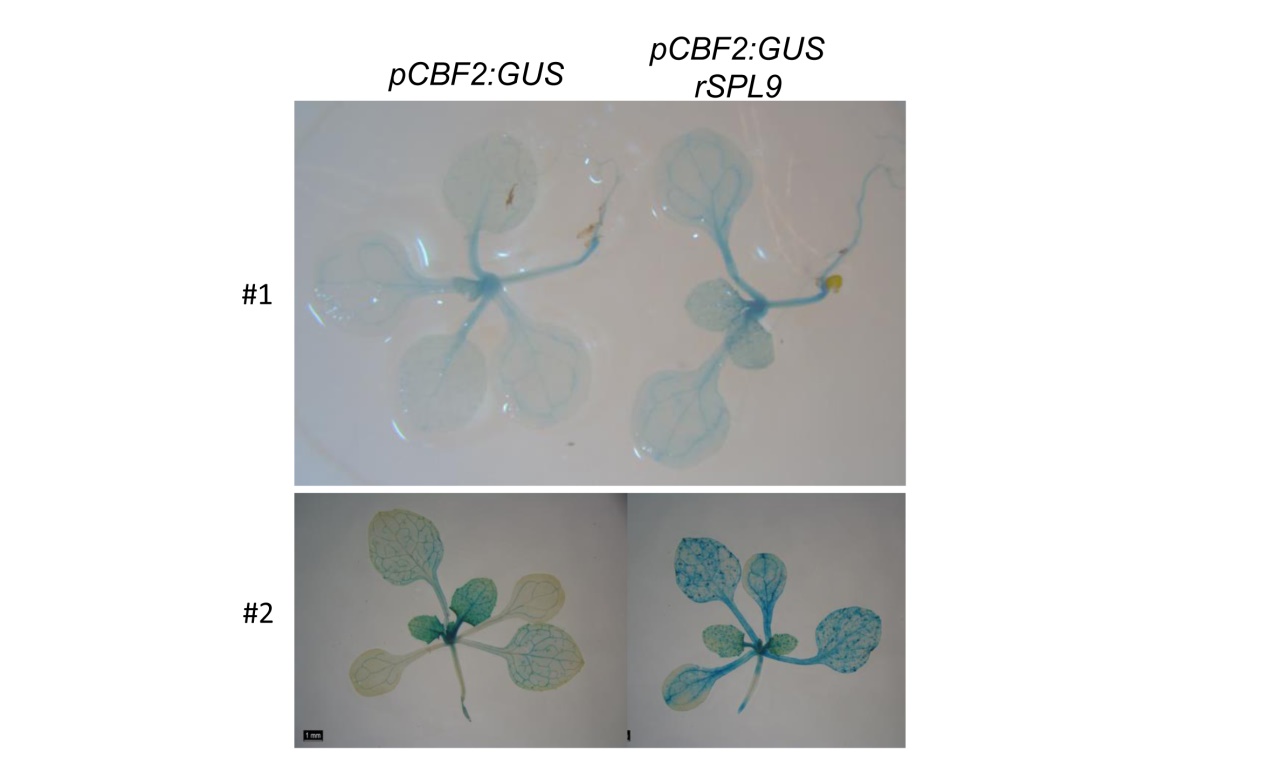


**Figure S7** GUS activity by histochemical staining in *pCBF2:GUS* and *rSPL9 pCBF2:GUS* plants*.*

*
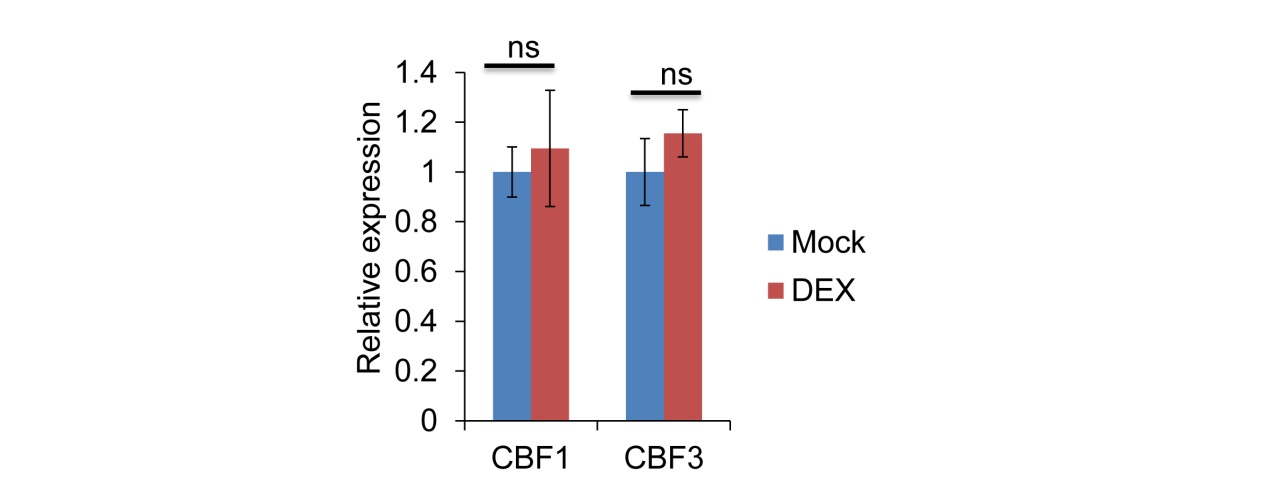
*

**Figure S8** RT-qPCR analysis of *CBF1* and *CBF3* expression, respectively, after SPL9 activation in the presence of DEX. ns, not significant.


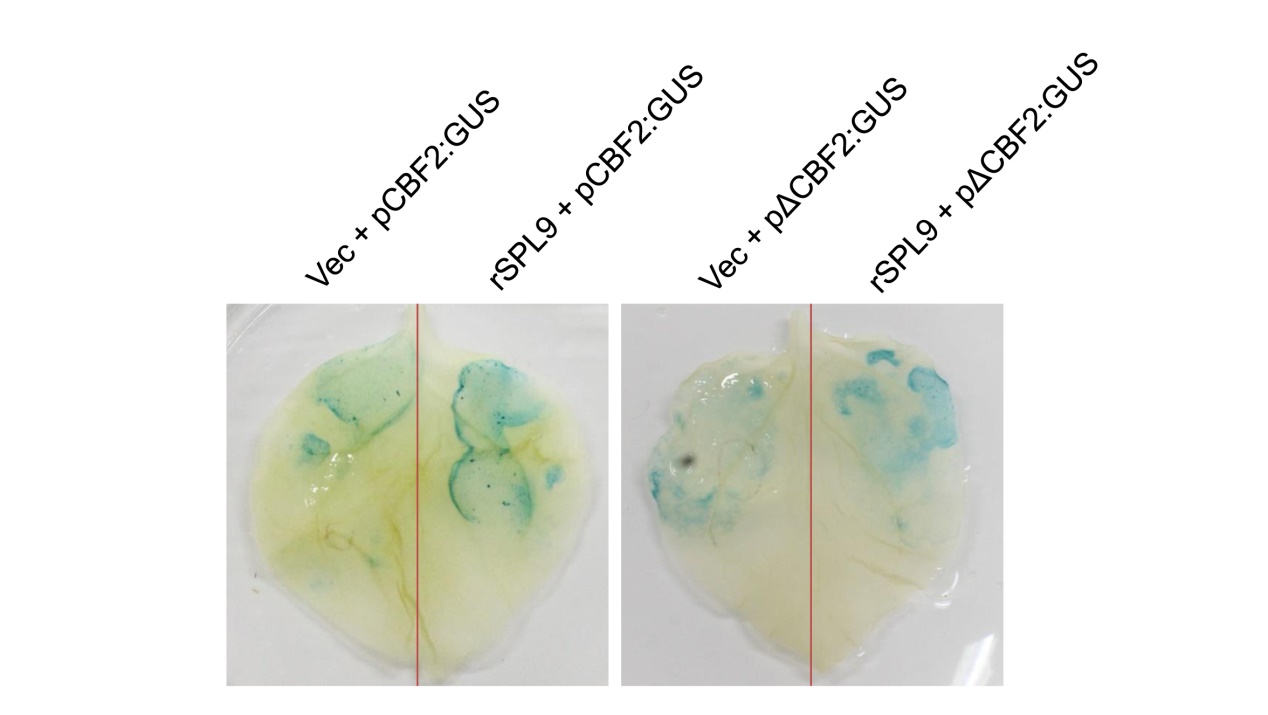


**Figure S9** GUS activity by histochemical staining in leaves of *N. benthamiana* infiltrated with *Agrobacterium* with different combinations of constructs, including Vec + pCBF2:GUS, Vec + pΔCBF2:GUS, rSPL9 + pCBF2:GUS, and rSPL9 + pΔCBF2:GUS.


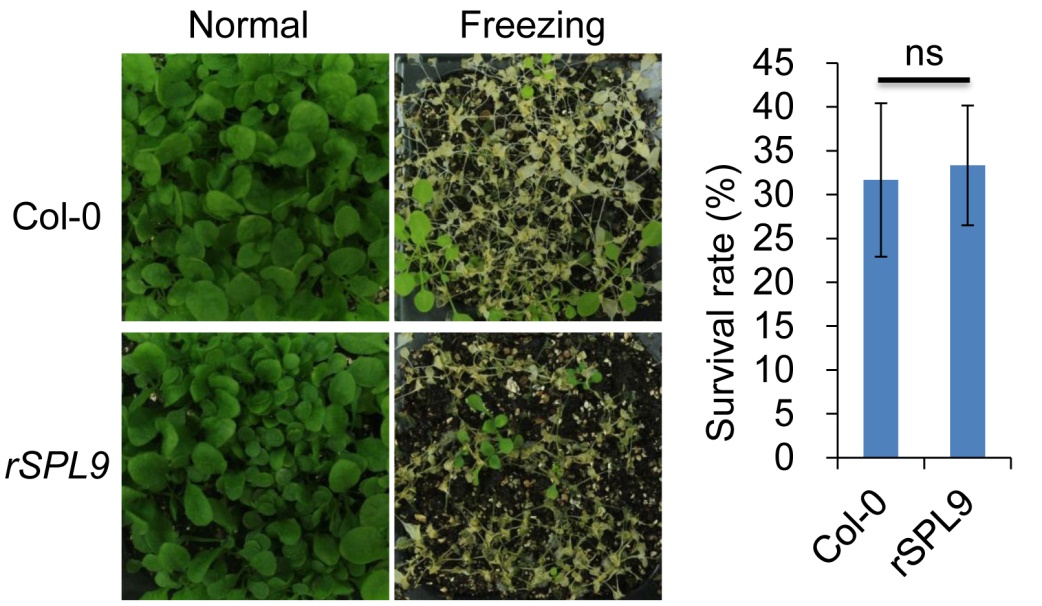


**Figure S10** Freezing phenotypes and survival rates of Col-0 and *rSPL9* plants after cold-acclimation treatment. ns, not significant.

**Table S1** Primers used in this study.

| Primer name | Sequence （5’ to 3’） | Note |
| --- | --- | --- |
| qTUB2-F | GAGCCTTACAACGCTACTCTGTCTGTC | RT-qPCR |
| qTUB2-R | ACACCAGACATAGTAGCAGAAATCAAG |  |
| qmiR172B-F | TTTCTCAAGCTTTAGGTATTTGTAG |  |
| qmiR172B-R | TCGGCGGATCCATGGAAGAAAGCTC |  |
| qSPL9-F | GGAATTTGACCTAGAGAAAAGGAGTT |  |
| qSPL9-R | GGGTTTTCAAGGTAGCAAATTGCT |  |
| qCBF2-F | TGACGTGTCCTTATGGAGCTA |  |
| qCBF2-R | CTGCACTCAAAAACATTTGCA |  |
| qSPL3-F | ATGAGTATGAGAAGAAGCAAAGCG |  |
| qSPL3-R | TCCACTACTACTTGTAGCTTTACCT |  |
| qSPL13-F | GGGTTTTCAAGGTAGCAAATTGCT |  |
| qSPL13-R | ACCAACAACATAGCTCTGGCTCTG |  |
| qKIN1-F | CCACATCTCTTCTCATCATCACTAAC |  |
| qKIN1-R | AAGGCATTCTTGTTGGTCTCTG |  |
| qRD29A-F | TGACGACGAAGTTACCTATCTCC |  |
| qRD29A-R | TCTCCGCCACATAATCTCTACC |  |
| qCOR47-F | GCCAAGACCACTGAAGAGGAAG |  |
| qCOR47-R | CAATCAACGAAAGCCACAATAACAACT |  |
| qWRKY6-F | CACTCACCTCCGCCTCCTAAT |  |
| qWRKY6-R | GTAAGTTCGTCATTTGCTGTTGTTGTTG |  |
| qSAG21-F | CGGCTGTGATGAAGAAGAAGG |  |
| qSAG21-R | GCGTCAATCTCGTTGGAACC |  |
| qSOC1-F | ACTGAGAGTGTATAAGGACAAGTAAC |  |
| qSOC1-R | GAGAGGCAAGTGTAAGAACATAGAT |  |
| qmiR156a-F | CTCTCCCTCCCTCTCTTTGATTC |  |
| qmiR156a-R | AGGCCAAAGAGATCAGCACCGG |  |
| qmiR156c-F | ATGAGTGATGACTGATGAGGGAGT |  |
| qmiR156c-R | GAGAAAGTGAGAGATGGGAACACA |  |
| qCBF1-F | TGTGATACGACGACCACGAAT |  |
| qCBF1-R | GCCTTCAGCCATATTATCCAACAA |  |
| qCBF3-F | CCTCAGGCGGTGATTATATTCC |  |
| qCBF3-R | CGACGAACTCCTCTGTATATTGG |  |
| snoR101-F | CTTCACAGGTAAGTTCGCTTG |  |
| snoR101-R | AGCATCAGCAGACCAGTAGTT |  |
| qmiR156-F | GCGGCGGTGACAGAAGAGAGT |  |
| Universal R | GTGCAGGGTCCGAGGT |  |
| miR156 RT | GTCGTATCCAGTGCAGGGTCCGAGGTATTCGCACTGGATACGACGTGCTC |  |
| ChIP-S1-F | AGCGAAGCGGCTACTGCATT | ChIP-qPCR |
| ChIP-S1-R | TAACTTAGTCCGTGACATCCCT |  |
| ChIP-eIF4A1-F | CACGCCCTGGAGTTCCAACAAC |  |
| ChIP-eIF4A1-R | GCAAATTGAGAAGGTCATGAGG |  |
| ChIP-ACT2-F | CCGCTTTGAATTGTCTCGTTG |  |
| ChIP-ACT2-R | CTTCTCTGTCAAGTCGCCG |  |
| ChIP-CBF2-1-F | ATGGTTGTCGTACCCTAT |  |
| ChIP-CBF2-1-R | AAGTTTCTGACCCTTCAC |  |
| ChIP-CBF2-2-F | CATACCATACAAAAAAAGACAGAGA |  |
| ChIP-CBF2-2-R | TGATCAGAAGAGTACTCTGTTTCA |  |
| *pCBF2-GUS-F* | CGGAATTCCATGCAAAATGTCTTTTCGCCT | Vector construction |
| *pCBF2-GUS-R* | CATGCCATGGTGATCAGAAGAGTACTCTGTTTCAAG |  |
| *p*Δ*CBF2-GUS-OLF1* | ATATTTAATAATGGTTGTCTGCACC |  |
| *p*Δ*CBF2-GUS-OLR1* | GTTTATAGGTGCAGACAACCATTATT |  |
| *p*Δ*CBF2-GUS-OLF2* | CGGAATTCCATGCAAAATGTCTTTTCGCCT |  |
| *p*Δ*CBF2-GUS-OLR2* | CATGCCATGGTGATCAGAAGATGCATCTGTTTCAAG |  |
